# How do immigration background, racism, socioeconomic circumstances and gender intersect to influence adolescent mental well-being? A longitudinal MAIHDA analysis of the multiethnic DASH cohort in London, UK

**DOI:** 10.1136/bmjph-2025-003283

**Published:** 2026-10-01

**Authors:** Alexis Karamanos, Divya Parmar, Jamie Murdoch, Paola Dazzan, Chiara Nosarti, Salma Ayis, Mariam Molokhia, Seeromanie Harding

**Affiliations:** 1 Population Health Sciences, King’s College London School of Population Health and Environmental Sciences, London, UK; 2 King’s College London Institute of Psychiatry Psychology & Neuroscience, London, UK

**Keywords:** Adolescent, Community Health, Epidemiology, Mental Health, Epidemiologic Methods

## Abstract

**Introduction:**

Despite increasing awareness of ethnic inequalities in mental health, UK-based research on the mental health experiences of adolescent migrants remains limited. This gap is particularly notable given the backdrop of rising legal migration and increasingly politicised public narratives surrounding immigration. Drawing on intersectionality theory, this study examines adolescent mental well-being at the intersections of immigration background, racism, socioeconomic disadvantage and gender.

**Methods:**

The British Determinants of Adolescent Social Well-being and Health longitudinal study followed up an ethnically diverse sample of 11–13-year-olds in London, UK, between 2002–2003 and 2005–2006. Using Multilevel Analysis of Individual Heterogeneity and Discriminatory Accuracy, we assessed the interplay between immigration background, racism, socioeconomic circumstances and gender using information on 4775 participants.

**Results:**

Notable intersectional inequalities involving immigration background, racism, socioeconomic disadvantage and gender were evident, regardless of age and ethnicity. These included UK-born males who reported racism and were least disadvantaged had higher predicted conduct symptom scores than foreign-born males who reported racism and were most disadvantaged. The lowest predicted conduct scores were among foreign-born females who did not report racism and were least disadvantaged (b=1.97, 95% CI 1.85 to 2.08). For hyperactivity, UK-born females who reported racism and were more disadvantaged had significantly higher scores than foreign-born males who did report racism and were more disadvantaged. The lowest hyperactivity scores were among foreign-born males who had not reported racism and were least disadvantaged (b=3.11, 95% CI 2.98 to 3.23). For internalising symptoms, foreign-born males who reported racism and were least disadvantaged had the highest peer relationship scores (b=2.46, 95% CI 2.35 to 2.58). Across all strata, females who reported racism had higher emotional symptom scores than those who did not, regardless of immigration status or socioeconomic circumstances.

**Conclusions:**

To fully understand inequalities in migrant adolescent mental well-being, the interplay between racism, socioeconomic disadvantage and gender must be explored.

**WHAT IS ALREADY KNOWN ON THIS TOPIC**

- Adolescent mental health inequalities linked to ethnicity are well established, yet the unique experiences of migrant youth in the UK remain underexplored. Existing studies often overlook how racism, poverty and gender combine to influence mental well-being.

**WHAT THIS STUDY ADDS**

- This study reveals that mental health symptoms in adolescents are shaped not just by one factor but by the complex intersections of immigration background, racism, socioeconomic disadvantage and gender.

**HOW THIS STUDY MIGHT AFFECT RESEARCH, PRACTICE OR POLICY**

- Our findings call for a shift in how adolescent mental health is studied and addressed—highlighting the urgent need for policies and interventions that recognise and respond to the lived realities of migrant youth facing racism and hardship.

## Background

 Adolescence is a formative stage of life during which key social and emotional skills—initially shaped in early childhood—are further developed. These skills play a foundational role in mental health across the life course. Adolescence also represents a crucial developmental period for the emergence of mental health problems, with an estimated 34.6% of individuals experiencing the onset of any mental disorder before the age of 14.^1^ Globally, mental disorders affect approximately one in seven adolescents aged 10–19, accounting for 15% of the total disease burden within this age group.^2^ This burden is increasing, particularly in the aftermath of the COVID-19 pandemic.^3^ Depression, anxiety and behavioural conditions are among the most prevalent contributors to ill health and disability during this period.

In the UK, mental health concerns among young people are rising. According to the *Mental Health of Children and Young People in England* report (2023), 20.3% of children aged 8–16 were identified as having both parent-reported and self-reported probable mental disorder, nearly doubling from 10.8% in 2017.^3^ Ethnic inequalities in adolescent mental health have been well documented. While certain ethnic minority groups appear to report better mental health outcomes than their white British counterparts,^4,5^ black Caribbean adolescents consistently experience disproportionately higher conduct problem symptoms than their white British counterparts.^6,7^ These variations, in part, may be explained by protective sociocultural factors, including stronger family and community networks, religious involvement and integration across ethnic groups.^5,8^

At the same time, the demographic profile of the UK is becoming increasingly diverse. Data from the 2021 UK Census shows that 16.8% of the population in England and Wales were born outside the UK, up from 13.4% in 2011—an increase that reflects broader trends in migration. Across Europe, mental health of adolescents with a migration background has become a growing area of research interest, as it is frequently viewed as an important measure of social integration. However, findings remain inconclusive. Some findings suggest a so-called ‘immigrant health advantage’, with migrant adolescents showing lower rates of mental health disorders compared with their native-born peers.^9^ Others highlight elevated risks of mental distress, including higher prevalence of depression, anxiety, sleep disturbances and psychotic symptoms among migrant youth.^10,11^

Despite increasing awareness of ethnic inequalities in mental health, relatively few UK-based studies have specifically examined the mental health experiences of adolescent migrants. This gap is particularly notable given the backdrop of rising legal migration and increasingly politicised public narratives surrounding immigration. The intersection of racism and immigration status is critical to understanding these inequalities. Migrant adolescents often face unique challenges that compound the effects of racism, including cultural dislocation and language barriers.^12^ Additionally, socioeconomic disadvantages such as living in deprived neighbourhoods and experiencing financial instability may further compound these challenges. The intersection of socioeconomic disadvantage with racism and immigration background creates a multifaceted vulnerability that can significantly influence adolescent mental health outcomes.

Emerging perspectives in social epidemiology and medical sociology underscore the importance of adopting intersectional approaches to understanding health inequalities.^13–16^ Nonetheless, much of the current literature continues to assess social determinants such as ethnicity, gender and socioeconomic circumstances (SECs) as isolated or additive factors.^17^ This reductive framing risks reinforcing deficit-based interpretations, where inequality is attributed to inherent group characteristics rather than broader structural and systemic processes.

Furthermore, traditional approaches often fail to account for the multiplicity and fluidity of individuals’ social positions over their life course. Many people simultaneously occupy advantaged and disadvantaged identities, which can shift depending on context.^18^ By focusing on between-group comparisons, such frameworks can also obscure important within-group variation, inadvertently treating marginalised populations as monolithic. Additionally, the continued use of dominant social groups (eg, white, male, affluent) as reference categories may sustain normative assumptions about who constitutes the standard, while sidelining the lived experiences of those at the intersections of privilege and marginalisation.

Intersectionality provides both a theoretical and methodological lens through which to examine how interlocking systems of power and inequality—such as race, gender, migration status, socioeconomic position and racism—collectively shape health outcomes.^19^ Unlike traditional single-axis approaches, intersectionality foregrounds the dynamic and mutually constitutive nature of social identities and structural conditions. One promising methodological tool aligned with this framework is Multilevel Analysis of Individual Heterogeneity and Discriminatory Accuracy (MAIHDA), which enables the quantitative modelling of intersectional complexity by simultaneously capturing within- and between-group variation.^20^ While inherently descriptive, MAIHDA offers valuable insights into how intersecting social positions contribute to health inequalities, serving as a critical complement to qualitative and participatory approaches within intersectional public health research.^21^

Despite growing interest in intersectionality, few studies on adolescent mental health explicitly adopt an intersectional framework. Most overlook the complex interplay of multiple social identities and structural factors—such as gender, immigration background and family socioeconomic conditions—and, notably, no existing study has incorporated direct measures of racism, because most observational studies have not collected data of experiences of discrimination. Furthermore, to our knowledge, the MAIHDA approach has yet to be employed to investigate adolescent mental health from an intersectional perspective within the UK context. This study uses the British Determinants of Adolescent Social Well-being and Health (DASH) study, known for its uniqueness in ethnic composition, to investigate adolescent mental well-being in relation to the intersections of immigration background, racism, socioeconomic disadvantage and gender. Our objective is to quantify how these dimensions intersect to shape emotional symptoms, peer relationship problems, hyperactivity/inattention and conduct symptoms in adolescence, by applying MAIHDA to the multiethnic DASH cohort in London.

## Methodology

### Design and sample

The DASH longitudinal study has been described in detail elsewhere.^22^ Briefly, between 2002 and 2003, a total of 6631 ethnically diverse students aged 11–13 years were recruited at baseline from 51 secondary schools across 10 London boroughs in the UK. A follow-up survey was conducted between 2005 and 2006 when participants were aged 14–16 years, achieving an 88% response rate among those invited.

Of the 4775 respondents who completed both waves, 948 were excluded from the complete-case analysis because of missing data in one or more key variables. Missingness was examined by comparing participants with complete data with those missing one or more analysis variables. Participants with missing data differed systematically from complete cases by sex, ethnicity, immigration background and reported racism, suggesting that the data were unlikely to be Missing Completely at Random (see online supplemental table 1).

### Dependent variables

Mental health outcomes were assessed using the self-reported Strengths and Difficulties Questionnaire (SDQ),^23^ a validated behavioural screening tool comprising multiple subscales. Each SDQ subscale consists of five items rated on a 3-point Likert scale: ‘Not True’, ‘Somewhat True’ and ‘Certainly True’. Responses were scored from 0 to 2 in accordance with the SDQ scoring guidelines. Subscales analysed included emotional symptoms and peer relationship problems (scores from these subscales are added to summarise broader internalising problem symptoms), hyperactivity/inattention and conduct symptoms (scores from these subscales are added to summarise broader externalising problem symptoms), with each generating a composite score ranging from 0 to 10. Higher scores indicate greater difficulties in the corresponding domain.

### Social strata dimensions

24 social strata were identified based on racism, family SECs, immigration background and gender by multiplying the number of their categories (2*3*2*2). Immigration background was determined based on self-reported country of birth (UK vs outside the UK). Gender was self-reported. In line with the original data collection, binary ‘male’ and ‘female’ categories were used in the analysis. Experiences of racism and discrimination were assessed at both baseline and follow-up using items from the Experiences of Discrimination scale.^24^ For the purposes of defining intersectional strata, we coded racism as ‘any experience during the study period’ vs ‘none’: if a participant reported at least one instance of unfair treatment (eg, due to race, skin colour, place of birth or religion) in a range of contexts (eg, school, public spaces) at either wave, they were classified as having experienced racism; otherwise, they were classified as no racism.

SECs were assessed using 17 binary (yes/no) items related to material standard of living, consistent with the approach used in a previous DASH study.^25^ These items captured indicators such as car ownership, housing conditions and access to household amenities, providing a composite measure of material deprivation, and they were categorised into tertiles based on thresholds derived from the baseline distribution across the full longitudinal sample.

### Controls

Previous research has identified age as a significant predictor of mental health outcomes during adolescence, with symptom trajectories typically fluctuating across this period.^26,27^ At each wave, age was calculated based on reported date of birth and the date of interview.

To facilitate the interpretation of model intercepts, age was mean-centred such that a value of zero corresponds to the sample’s median age. Age was included as a time-varying fixed effect. At each wave, it was calculated based on reported date of birth and the date of interview. Random slopes for wave were considered, but models including a random slope for wave at the stratum level produced singular fits, near-zero slope variance and boundary correlations, indicating that the data did not support differential trajectories across intersectional strata. We therefore retained a random-intercept-only specification as the more parsimonious and better-identified model.

Ethnicity was self-reported and cross-validated using parental ethnicity and grandparents’ countries of birth. Ethnic categories offered used the England Census categories. Participants were categorised into the following ethnic groups: white UK, black Caribbean, black African, Indian, Pakistani/Bangladeshi and Other. The ‘Other’ category included individuals identifying as Mixed, Irish, Greek, Turkish, Kosovan, Albanian, Kurdish, Vietnamese, Singaporean, Filipino, Japanese, Chinese and other Asian backgrounds.

### Missing data

Missing data were low for SDQ outcomes (<2%) but higher for experience of racism (25%) and SECs (14%). Multiple Imputation by Chained Equations was therefore used under the Missing At Random assumption to reduce potential bias and avoid excluding participants with partial data.^28^

25 imputed datasets were generated. The imputation model included all analysis variables across both waves, including sex, ethnicity, immigration background, SECs, racism, SDQ subscales, chronological age and auxiliary air pollution variables (modelled concentrations of NO_₂_, O_₃_, PM_₁₀_ and PM_₂.₅_) to help predict missing data. Imputed values were checked for plausibility, and estimates were combined using Rubin’s rules.^29^ Imputed values were checked using convergence diagnostics and distributional comparisons.

Descriptive results are presented for both complete-case and imputed data. For the MAIHDA analyses, following recommendations, imputed SDQ outcome values were excluded to avoid potential issues associated with imputed outcomes, leaving 4694 observations.^30^

### Statistical analysis

All MAIHDA models were implemented in R V.4.2.3 using the lme4 package. Linear mixed-effects models were fitted using restricted maximum likelihood estimation.

To account for the longitudinal structure of the data, we extended the conventional two-level MAIHDA framework by modelling repeated measurement occasions as level 1. The resulting structure comprised measurement occasions nested within individuals (level 2), with individuals grouped within intersectional strata defined by stable, time-invariant social characteristics (level 3).

Because schools were not nested within intersectional strata, we included schools as crossed random effects (level 3). This allowed for the fact that the same intersectional stratum could include students from multiple schools, while each school could include students from multiple intersectional strata.

Two models were specified: (Model A) a null model including random intercepts only, and (Model B) a main-effects model controlling for age and ethnicity. For each model, we calculated the variance partition coefficient (VPC) to estimate the proportion of outcome variance attributable to individual-level, stratum-level and school-level differences.

The VPC was interpreted as a measure of discriminatory accuracy—indicating the model’s ability to differentiate between individuals based on intersectional group membership. We also estimated the proportional change in variance (PCV) to assess the reduction in between-stratum variance from the null to the main-effects model.

Predicted marginal means were derived from the main-effects model (Model B). To quantify uncertainty, we employed parametric bootstrapping using the bootMer function with 1000 resampled model fits. CIs (95%) were obtained from the 2.5th and 97.5th percentiles of the bootstrapped prediction distributions, capturing variability across simulations.

### Patient and public involvement

DASH was supported by teachers, parents and community leaders during conceptualisation, pilot, data collection phases and disseminating of results in schools and newsletters.

## Results

In the imputed dataset (table 1), more than one in four DASH participants were born outside the UK (27.8%). The mean age of participants across the two study waves was 13.9 years. Compared with UK-born participants, foreign-born participants had significantly lower mean scores for conduct symptoms but significantly higher levels of peer relationship problems. More than one-third of foreign-born participants identified as belonging to other minority ethnic groups (36.4%), compared with 17.5% of UK-born participants. Among foreign-born participants, 25.6% were black African, compared with 14.6% of UK-born participants. Foreign-born participants were more likely to be classified as least disadvantaged by family SEC, but were also significantly more likely to report experiences of racism during the study period than UK-born participants (34.4% vs 27.7%, p<0.001).

**Table 1:** Descriptive profile of sample by immigration background for British Determinants of Adolescent Social Well-being and Health participants aged 11–16 years (N=4775)

Characteristic	UK-born (72.2%)	Born abroad (27.8%)	P value
Age (years)	13.9 (1.4)	13.9 (1.5)	0.048
Emotional symptoms	2.7 (2.1)	2.8 (2.1)	0.088
Peer problems	1.7 (1.5)	2.1 (1.6)	<0.001
Conduct problems	2.4 (1.7)	2.3 (1.6)	<0.001
Hyperactivity	3.8 (2.2)	3.3 (2.1)	0.207
Sex			<0.001
Female	47.1%	40.8%	
Male	52.9%	59.2%	
Ethnicity			<0.001
Other	17.5%	36.4%	
Black Caribbean	15.1%	14.5%	
Black African	14.6%	25.6%	
Indian	8.4%	8.0%	
Mixed	9.8%	5.0%	
Pakistani and Bangladeshi	10.0%	8.7%	
White UK	24.6%	1.9%	
Socioeconomic disadvantage tertile			<0.001
Least disadvantaged tertile	41.8%	56.5%	
Middle tertile	31.1%	28.0%	
Most disadvantaged tertile	27.0%	15.5%	
Experienced racism			<0.001
No racism	72.2%	65.6%	
Racism	27.8%	34.4%	

Continuous variables: mean (SD). Categorical variables: N (%). p-values pooled using Rubin’s rules: t-test for continuous variables, Li–Raghunathan D2 statistic for categorical variables.

Results from the MAIHDA models, shown in online supplemental tables 2–4, indicated between-stratum variation in all four mental health outcomes assessed. In the null models (Model A), most variance was attributable to differences between and within DASH participants, while less than 2% of the variance was attributable to schools for all outcomes. Online supplemental table 4 highlights that the proportion of variance attributable to intersectional strata was 3.8% for peer relationship problems, 3.1% for conduct symptoms and 2.2% for hyperactivity/inattention symptoms. Emotional symptoms showed a higher intersectional stratum-level VPC of 10.3%. These values represent the proportion of total variance attributable to differences between intersectional strata before accounting for additive fixed effects, age and ethnicity. Although these VPCs were modest, they are consistent with the magnitudes typically observed in multilevel models that nest individuals within social contexts.^28^

After adjustment for additive main effects, age and ethnicity (Model B), the PCV indicated that most of the between-stratum variance was explained by these covariates. PCV values were 100% for emotional symptoms, 96.5% for hyperactivity/inattention, 95.8% for peer relationship problems and 93.8% for conduct symptoms. A PCV of 100% indicates that no residual between-stratum variance remained after accounting for additive effects. The small residual variance observed for conduct, hyperactivity/inattention and peer relationship problems suggests limited remaining intersectional patterning beyond the additive effects included in the model.

Predicted mean scores and corresponding 95% CIs were calculated for each stratum (figures 1–4 and online supplemental tables 5–8). Despite relatively small between-stratum variance, the predicted outcomes revealed intersectional inequalities. UK-born males who reported racism and were most disadvantaged had higher predicted conduct symptom scores (b=3.03, 95% CI 2.90 to 3.16) than foreign-born males who reported racism and were least disadvantaged (b=2.65, 95% CI 2.51 to 2.79) (figure 1 and online supplemental table 5). The lowest predicted conduct scores were among foreign-born females who did not report racism and were least disadvantaged (b=1.97, 95% CI 1.85 to 2.08). For hyperactivity/inattention (figure 2 and online supplemental table 6), UK-born females who reported racism and were more disadvantaged had significantly higher scores than foreign-born males who did report racism and were more disadvantaged. The lowest hyperactivity scores were among foreign-born males who had not reported racism and were least disadvantaged (b=3.11, 95% CI 2.98 to 3.23). For internalising symptoms, foreign-born males who reported racism and were least disadvantaged had the highest peer relationship scores (b=2.46, 95% CI 2.35 to 2.58) (figure 3 and online supplemental table 7). Across all strata, females who reported racism had higher emotional symptom scores than those who did not, regardless of immigration status or SECs (figure 4 and online supplemental table 8). In terms of additive associations (see online supplemental table 2), an increase in age was significantly associated with lower emotional, peer relationship and conduct symptom scores, but to higher hyperactivity/inattention scores. Participants who reported experiences of racism consistently had higher peer relationship, emotional, hyperactivity and conduct symptom scores than those who did not. Foreign-born participants had significantly higher peer relationship and emotional symptom scores, yet lower conduct and hyperactivity/inattention scores, relative to their UK-born counterparts. Female participants exhibited significantly higher adjusted emotional symptom scores but lower conduct symptom scores. Participants from minority ethnic backgrounds generally had lower symptom scores compared with white British participants, except for those from mixed ethnic backgrounds, who had higher conduct symptom scores.

**Figure 1:**
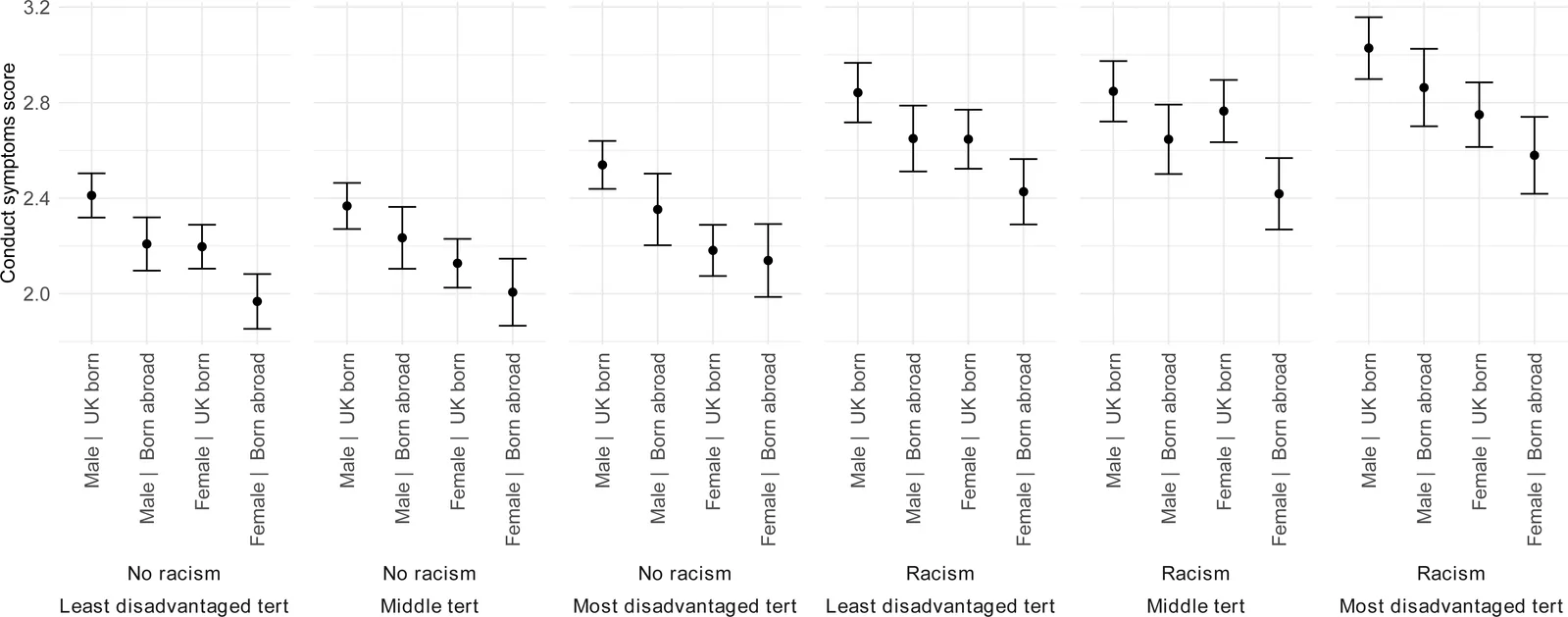
Conduct symptoms and intersectional patterns by immigration background, racism, socioeconomic circumstances and gender: Determinants of Adolescent Social Well−being and Health.

**Figure 2:**
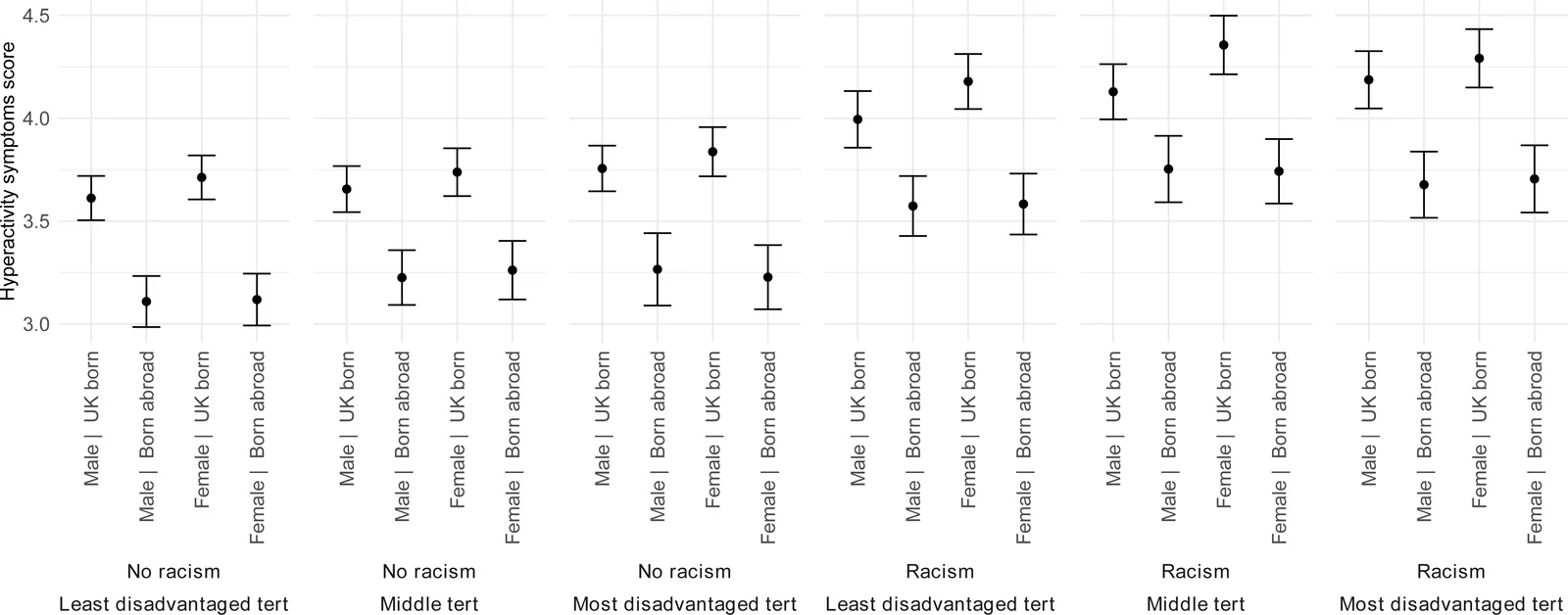
Hyperactivity symptoms and intersectional patterns by immigration background, racism, socioeconomic circumstances and gender: Determinants of Adolescent Social Well−being and Health.

**Figure 3:**
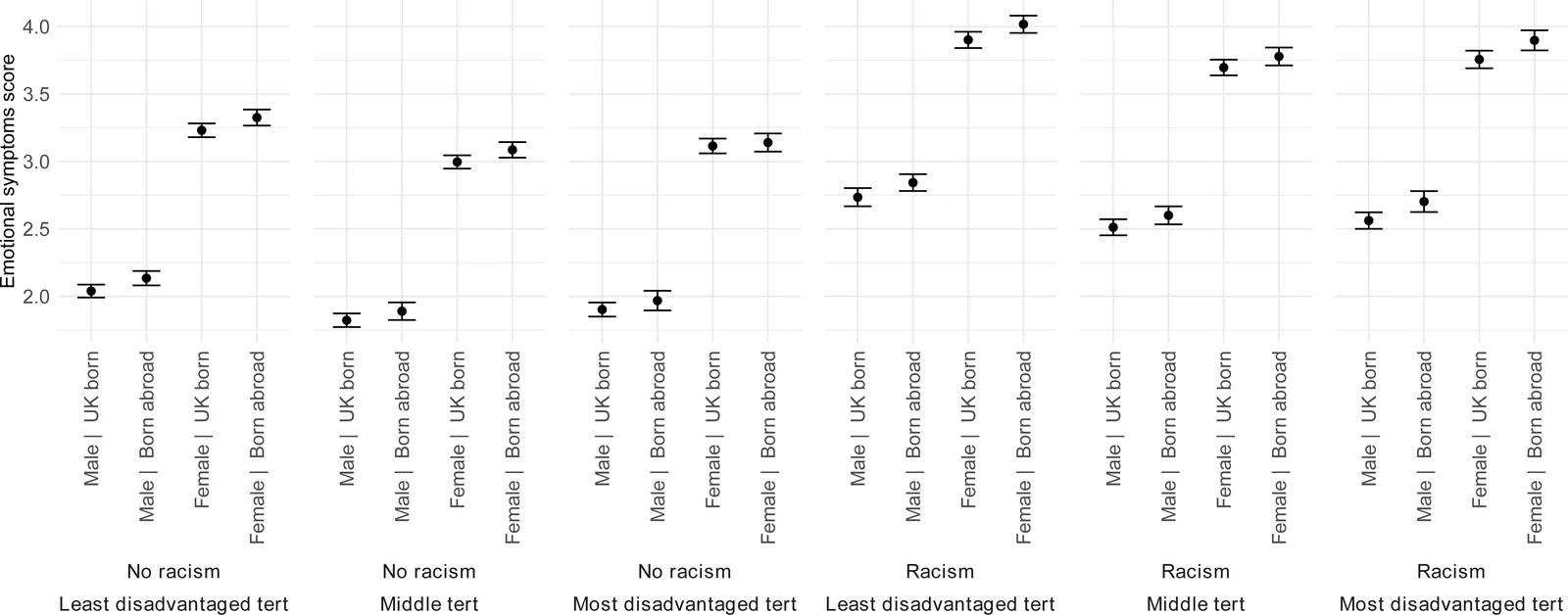
Emotional symptoms and intersectional patterns by immigration background, racism, socioeconomic circumstances and gender: Determinants of Adolescent Social Well−being and Health.

**Figure 4:**
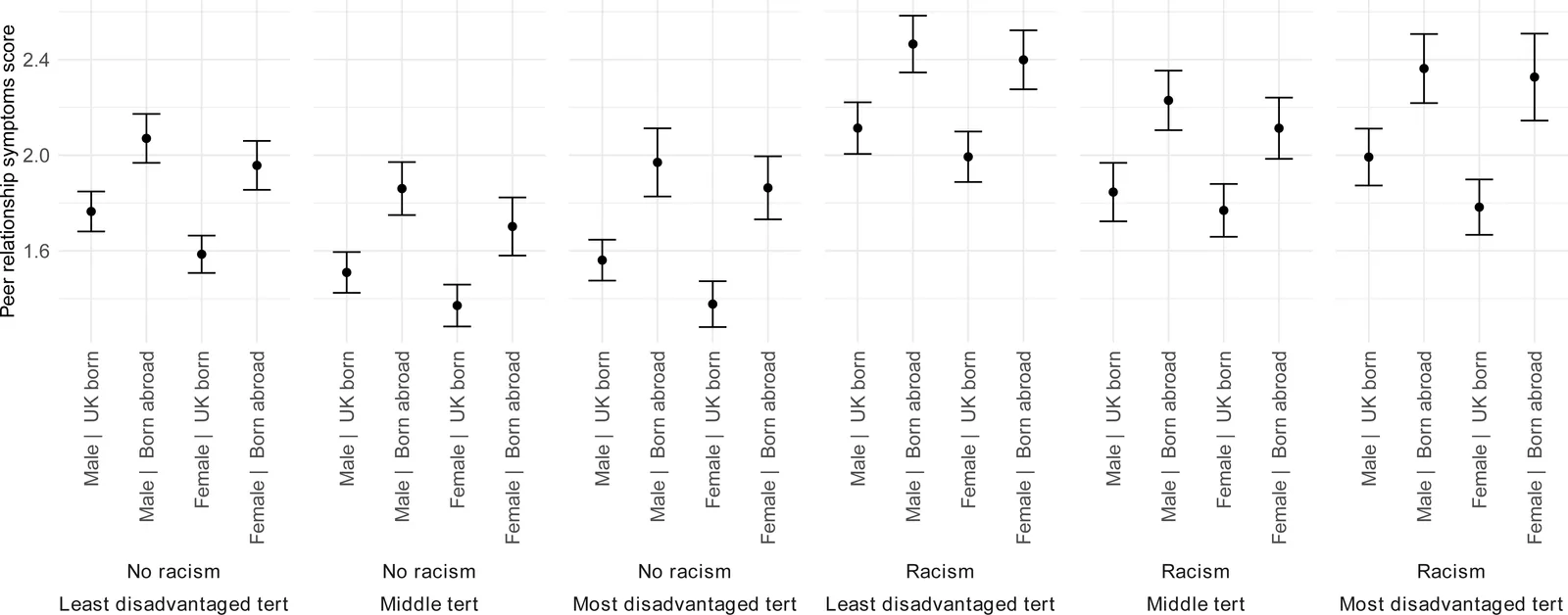
Peer Relationship symptoms and intersectional patterns by immigration background, racism, socioeconomic circumstances and gender: Determinants of Adolescent Social Well-being and Health.

## Discussion

The present study provides significant insights into the mental health outcomes of a diverse cohort of adolescents in London, with a particular focus at the intersections of immigration background, SEC, racism and gender. Our findings corroborate previous research, as foreign-born participants reported significantly lower mean scores for conduct and hyperactivity/inattention symptoms compared with UK-born counterparts, suggesting fewer behavioural problems among migrant adolescents.^31,32^ However, they also reported higher levels of emotional symptoms and peer relationship problems, pointing to the social and psychological challenges that may accompany migration and minoritised status. A higher proportion of foreign-born participants identified as belonging to minority ethnic groups and reported experiences of racism. The higher prevalence of reported racism among foreign-born participants, together with its association with higher symptom scores across all mental health outcomes, highlights racism as an important determinant of adolescent mental health.^33,34^ Predicted mean scores did not provide clear evidence that socioeconomic advantage buffered the psychological impact of racism.

The study’s findings suggest several actionable implications for policy and practice. First, antiracism prevention and response should be embedded within schools and youth services through routine surveillance of discriminatory incidents, clear reporting and support pathways, staff training in culturally safe, trauma-informed practice and partnerships with community organisations, given consistent evidence that exposure to racism is associated with poorer mental health across multiple domains.^33,35^

Second, given elevated peer difficulties among foreign-born adolescents, targeted social-integration supports—including structured peer mentoring, focused anti-bullying initiatives should be prioritised in settings with higher proportions of migrant pupils, with progress monitored using co-designed indicators of belonging and connectedness. Finally, for subgroups in which conduct and hyperactivity/inattention difficulties co-occur with exposure to racism and socio-economic hardship, early, evidence-informed behavioural supports (eg, classroom self-regulation strategies, small-group coaching) should be coupled with family-centred material assistance (benefits advice, housing and food-security referrals) and coordinated across education, health and community services. A notable strength of this study is its foundation in the DASH study, a large multiethnic cohort of adolescents in London. The DASH study’s comprehensive data collection, including validated measures of experiences of discrimination, has facilitated this intersectional analysis. This is particularly noteworthy as other UK national and regional cohort studies, such as the Avon Longitudinal Study of Parents and Children,^36^ Next Steps^37^ and Millennium Cohort Study,^38^ lack data on experiences of discrimination, thereby limiting their capacity to conduct similar analyses. The utilisation of MAIHDA models represents another strength of this study. MAIHDA offers a clearer interpretation of intersectional effects by focusing on the variance attributable to different strata (eg, combinations of social identities) rather than individual interaction terms.^39^ This approach enhances our understanding of the combined impact of multiple social determinants on adolescent mental health outcomes. Furthermore, MAIHDA can more effectively manage small sample sizes within intersectional strata, thereby increasing the statistical power to detect meaningful differences between groups, which might be overlooked in traditional regression models with multiple interactions.

This study has several limitations. First, the binary measurement of racism, though analytically convenient, oversimplifies the frequency, severity and context of racism experiences, potentially underestimating their cumulative impact on adolescent mental health. Second, the conflation of sex and gender in the binary response options reflects outdated measurement practices and limits the scope for gender-sensitive analyses. Third, the use of tertiles to describe family SEC, while practical, reduces a complex construct to a narrow classification, overlooking critical dimensions such as housing insecurity and material deprivation that may independently affect mental health.

The ethnicity classification may not fully capture the heterogeneity and nuance within individual ethnic groups, particularly among participants categorised as mixed or other ethnic backgrounds. Although the analysis used repeated observations from two waves, it was not designed to estimate developmental trajectories. Age was included as a fixed effect, but random slopes for wave were not retained because sensitivity models produced singular fits and near-zero slope variance. The findings should therefore be interpreted as age-adjusted longitudinal associations, rather than evidence of within-person developmental change over time. Finally, while the DASH cohort provides valuable insight into the experiences of urban, multiethnic adolescents, its focus on London may limit the generalisability of the findings to young people in other regional or national contexts.

## Supplementary material

10.1136/bmjph-2025-003283 online supplemental table 1

## Data Availability

Data are available upon reasonable request.
