# Supplementary material for: How do immigration background, racism, socioeconomic circumstances and gender intersect to influence adolescent mental well-being? A longitudinal MAIHDA analysis of the multiethnic DASH cohort in London, UK
Source: BMJ Public Health. 2026 Oct 1;4(4):e003283. doi: 10.1136/bmjph-2025-003283 (PMC13663703; doi:10.1136/bmjph-2025-003283)
Supplement: Supplementary file 1 [file bmjph-4-4-s001.docx]

**Supplementary statistical tables**

**Supplementary table 1. Comparison of complete cases and participants with missing data**

| **Characteristic** | **Complete cases** | **Missing data** | **p value** |
| --- | --- | --- | --- |
| **Sex** |  |  |  |
| Male | 52.0% | 60.4% | <0.001 |
| Female | 48.0% | 39.6% |  |
| **Ethnicity** |  |  |  |
| White UK | 21.8% | 10.6% | <0.001 |
| Black Caribbean | 14.0% | 16.9% |  |
| Black African | 15.8% | 21.6% |  |
| Indian | 8.6% | 7.6% |  |
| Pakistani & Bangladeshi | 9.8% | 9.2% |  |
| Mixed | 8.4% | 8.7% |  |
| All Others | 21.6% | 25.3% |  |
| **Birth status / immigration variable** |  |  |  |
| UK born | 79.5% | 56.0% | <0.001 |
| Born abroad | 20.5% | 44% |  |
| **Disadvantage, wave 1** |  |  |  |
| Least disadvantaged | 45.2% | 53.2% | <0.001 |
| Middle tertile | 21.7% | 20.3% |  |
| Most disadvantaged | 33.1% | 26.5% |  |
| **Disadvantage, wave 2** |  |  |  |
| Least disadvantaged | 40.9% | 50.7% | <0.001 |
| Middle tertile | 41.1% | 35.2% |  |
| Most disadvantaged | 18.0% | 14.1% |  |
|  |  |  |  |
| **Racism, wave 1** |  |  |  |
| No racism | 73.2% | 70.1% | <0.001 |
| Racism | 26.8% | 29.9% |  |
| **Racism, wave 2** |  |  |  |
| No racism | 68.3% | 66.4% | <0.001 |
| Racism | 31.7% | 33.6% |  |
| **Emotional symptoms, wave 1** |  |  |  |
|  | 2.8 (2.1) | 2.9 (2.3) | 0.064 |
| **Emotional symptoms, wave 2** |  |  |  |
|  | 2.6 (2.0) | 2..7 (2.0) | 0.169 |
| **Conduct problems, wave 1** |  |  |  |
|  | 2.4 (1.7) | 2.5 (1.7) | 0.013 |
| **Conduct problems, wave 2** |  |  |  |
|  | 2.3 (1.6) | 2.5 (1.7) | 0.002 |
| **Hyperactivity/inattention, wave 1** |  |  |  |
|  | 3.7 (2.2) | 3.6 (2.1) | 0.143 |
| **Hyperactivity/inattention, wave 2** |  |  |  |
|  | 3.8 (2.2) | 3.6 (2.0) | 0.002 |
| **Peer relationship problems, wave 1** |  |  |  |
|  | 1.8 (1.5) | 2.2 (1.7) | <0.001 |
| **Peer relationship problems, wave 2** |  |  |  |
|  | 1.5 (1.4) | 1.8 (1.6) | <0.001 |
| **Age, wave 1** |  |  |  |
|  | 12.6 (0.6) | 12.5 (0.6) | <0.001 |
| **Age, wave 2** |  |  |  |
|  | 15.2 (0.6) | 15.1 (0.6) | 0.001 |

*Note. Categorical variables are shown as percentages only; continuous variables are shown as mean (SD). P values were calculated using t-test and chi-square tests..*

**Multilevel linear regression models for SDQ outcomes**

**Supplementary Table 2. Fixed effects from multilevel linear regression models**

| **Parameter** | **Conduct symptoms β (95% CI)** | **p value** | **Emotional symptoms β (95% CI)** | **p value** | **Hyperactivity symptoms β (95% CI)** | **p value** | **Peer relationship symptoms β (95% CI)** | **p value** |
| --- | --- | --- | --- | --- | --- | --- | --- | --- |
| **Null model** |  |  |  |  |  |  |  |  |
| Intercept | 2.48 (2.34 to 2.62) | <0.001 | 2.90 (2.62 to 3.18) | <0.001 | 3.73 (3.57 to 3.89) | <0.001 | 1.90 (1.76 to 2.03) | <0.001 |
| **Main effects model** |  |  |  |  |  |  |  |  |
| Intercept | 2.75 (2.42 to 3.07) | <0.001 | 3.35 (2.96 to 3.73) | <0.001 | 3.40 (2.99 to 3.81) | <0.001 | 3.55 (3.25 to 3.86) | <0.001 |
| Age | -0.03 (-0.05 to -0.01) | 0.013 | -0.08 (-0.10 to -0.05) | <0.001 | 0.04 (0.01 to 0.07) | 0.003 | -0.13 (-0.15 to -0.11) | <0.001 |
| Born abroad (ref: UK born) | -0.21 (-0.32 to -0.10) | <0.001 | 0.15 (0.04 to 0.26) | 0.005 | -0.36 (-0.49 to -0.23) | <0.001 | 0.34 (0.24 to 0.44) | <0.001 |
| Female (ref: male) | -0.25 (-0.35 to -0.14) | <0.001 | 1.20 (1.11 to 1.29) | <0.001 | -0.03 (-0.15 to 0.09) | 0.676 | -0.07 (-0.16 to 0.02) | 0.136 |
| Middle tertile (ref: least disadvantaged) | 0.03 (-0.09 to 0.15) | 0.607 | -0.21 (-0.32 to -0.10) | <0.001 | 0.08 (-0.06 to 0.21) | 0.249 | -0.18 (-0.29 to -0.08) | <0.001 |
| Most disadvantaged tertile (ref: least disadvantaged) | 0.14 (0.01 to 0.27) | 0.029 | -0.20 (-0.33 to -0.08) | <0.001 | 0.10 (-0.05 to 0.24) | 0.202 | -0.20 (-0.31 to -0.08) | 0.001 |
| Experienced racism (ref: no racism) | 0.48 (0.37 to 0.59) | <0.001 | 0.70 (0.60 to 0.80) | <0.001 | 0.50 (0.38 to 0.62) | <0.001 | 0.42 (0.32 to 0.51) | <0.001 |
| Ethnicity: All other ethnic groups (ref: White UK) | 0.03 (-0.09 to 0.15) | 0.660 | -0.18 (-0.33 to -0.04) | 0.013 | -0.35 (-0.51 to -0.20) | <0.001 | 0.02 (-0.09 to 0.13) | 0.684 |
| Ethnicity: Black Caribbean (ref: White UK) | 0.27 (0.14 to 0.40) | <0.001 | -0.40 (-0.55 to -0.24) | <0.001 | -0.23 (-0.40 to -0.06) | 0.008 | -0.15 (-0.27 to -0.04) | 0.009 |
| Ethnicity: Black African (ref: White UK) | 0.09 (-0.04 to 0.21) | 0.200 | -0.49 (-0.64 to -0.34) | <0.001 | -0.58 (-0.75 to -0.41) | <0.001 | -0.22 (-0.34 to -0.10) | <0.001 |
| Ethnicity: Indian (ref: White UK) | -0.28 (-0.43 to -0.12) | <0.001 | -0.16 (-0.34 to 0.02) | 0.089 | -0.78 (-0.98 to -0.57) | <0.001 | -0.08 (-0.22 to 0.07) | 0.293 |
| Ethnicity: Mixed ethnicity (ref: White UK) | 0.25 (0.10 to 0.40) | 0.001 | -0.39 (-0.57 to -0.21) | <0.001 | 0.01 (-0.19 to 0.20) | 0.927 | -0.20 (-0.34 to -0.07) | 0.003 |
| Ethnicity: Pakistani/Bangladeshi (ref: White UK) | -0.23 (-0.38 to -0.08) | 0.003 | -0.10 (-0.27 to 0.08) | 0.292 | -0.75 (-0.95 to -0.55) | <0.001 | -0.05 (-0.19 to 0.08) | 0.453 |

*Values are β coefficients with 95% confidence intervals unless otherwise stated. P values are shown to three decimal places. Ref, reference category*

**Supplementary Table 3. Random-effects variance components**

| **Variance component** | **Conduct symptoms estimate (95% CI)** | **Emotional symptoms estimate (95% CI)** | **Hyperactivity symptoms estimate (95% CI)** | **Peer relationship symptoms estimate (95% CI)** |
| --- | --- | --- | --- | --- |
| **Null model** |  |  |  |  |
| Individual (intercept) | 1.15 (1.11 to 1.20) | 1.53 (1.48 to 1.58) | 2.12 (2.06 to 2.18) | 0.62 (0.57 to 0.67) |
| Residual | 1.55 (1.51 to 1.59) | 2.48 (2.44 to 2.52) | 2.42 (2.36 to 2.48) | 1.65 (1.60 to 1.70) |
| School (intercept) | 0.03 (0.03 to 0.03) | 0.01 (0.01 to 0.01) | 0.07 (0.07 to 0.08) | 0.03 (0.02 to 0.03) |
| Stratum (intercept) | 0.09 (0.07 to 0.10) | 0.46 (0.43 to 0.49) | 0.10 (0.09 to 0.12) | 0.09 (0.07 to 0.11) |
| **Main effects model** |  |  |  |  |
| Individual (intercept) | 1.14 (1.10 to 1.18) | 1.53 (1.48 to 1.57) | 2.08 (2.02 to 2.15) | 0.65 (0.60 to 0.70) |
| Residual | 1.55 (1.51 to 1.59) | 2.45 (2.41 to 2.49) | 2.40 (2.33 to 2.46) | 1.58 (1.54 to 1.63) |
| School (intercept) | 0.02 (0.02 to 0.02) | 0.01 (0.00 to 0.01) | 0.05 (0.05 to 0.06) | 0.02 (0.02 to 0.02) |
| Stratum (intercept) | 0.01 (0.00 to 0.01) | 0.00 (0.00 to 0.00) | 0.00 (0.00 to 0.01) | 0.00 (0.00 to 0.01) |

*Values are random-effect variance estimates with 95% confidence intervals. Estimates and confidence intervals are rounded to two decimal places.*

**Supplementary Table 4. Variance partition coefficients and proportional change in variance**

| **Measure** | **Conduct symptoms** | **Emotional symptoms** | **Hyperactivity symptoms** | **Peer relationship symptoms** |
| --- | --- | --- | --- | --- |
| **VPC (%)** |  |  |  |  |
| **Null model** |  |  |  |  |
| Individual | 40.85 | 34.10 | 44.94 | 26.12 |
| Residual | 54.94 | 55.28 | 51.28 | 68.97 |
| School | 1.10 | 0.29 | 1.55 | 1.11 |
| Stratum | 3.11 | 10.34 | 2.22 | 3.81 |
| **Main effects model** |  |  |  |  |
| Individual | 41.97 | 38.32 | 45.87 | 28.63 |
| Residual | 57.05 | 61.55 | 52.86 | 70.20 |
| School | 0.78 | 0.13 | 1.18 | 0.99 |
| Stratum | 0.20 | 0.00 | 0.08 | 0.17 |
| **PCV (%)** |  |  |  |  |
| Individual | 1.30 | 0.20 | 1.73 | -3.73 |
| Residual | 0.25 | 1.10 | 0.75 | 3.69 |
| School | 31.86 | 60.05 | 26.52 | 15.39 |
| Stratum | 93.82 | 99.96 | 96.54 | 95.78 |

*VPC, variance partition coefficient; PCV, proportional change in variance. VPC and PCV values are shown as percentages and rounded to two decimal places.*

**Supplementary Table 5. Marginal predicted means for conduct problems**

| **Sex** | **Birth status** | **Experience of racism** | **Disadvantage tertile** | **n** | **SE** | **Predicted mean (95% CI)** |
| --- | --- | --- | --- | --- | --- | --- |
| Male | UK born | Racism | Most disadvantaged | 283 | 0.07 | 3.03 (2.90 to 3.16) |
| Male | Born abroad | Racism | Most disadvantaged | 88 | 0.08 | 2.86 (2.70 to 3.03) |
| Male | UK born | Racism | Middle | 326 | 0.06 | 2.85 (2.72 to 2.97) |
| Male | UK born | Racism | Least disadvantaged | 435 | 0.06 | 2.84 (2.72 to 2.97) |
| Female | UK born | Racism | Middle | 299 | 0.07 | 2.76 (2.63 to 2.89) |
| Female | UK born | Racism | Most disadvantaged | 211 | 0.07 | 2.75 (2.61 to 2.88) |
| Male | Born abroad | Racism | Least disadvantaged | 291 | 0.07 | 2.65 (2.51 to 2.79) |
| Female | UK born | Racism | Least disadvantaged | 378 | 0.06 | 2.65 (2.52 to 2.77) |
| Male | Born abroad | Racism | Middle | 176 | 0.07 | 2.65 (2.50 to 2.79) |
| Female | Born abroad | Racism | Most disadvantaged | 38 | 0.08 | 2.58 (2.42 to 2.74) |
| Male | UK born | No racism | Most disadvantaged | 778 | 0.05 | 2.54 (2.44 to 2.64) |
| Female | Born abroad | Racism | Least disadvantaged | 218 | 0.07 | 2.43 (2.29 to 2.56) |
| Female | Born abroad | Racism | Middle | 98 | 0.08 | 2.42 (2.27 to 2.57) |
| Male | UK born | No racism | Least disadvantaged | 1010 | 0.05 | 2.41 (2.32 to 2.50) |
| Male | UK born | No racism | Middle | 818 | 0.05 | 2.37 (2.27 to 2.46) |
| Male | Born abroad | No racism | Most disadvantaged | 181 | 0.08 | 2.35 (2.20 to 2.50) |
| Male | Born abroad | No racism | Middle | 308 | 0.07 | 2.23 (2.10 to 2.36) |
| Male | Born abroad | No racism | Least disadvantaged | 524 | 0.06 | 2.21 (2.10 to 2.32) |
| Female | UK born | No racism | Least disadvantaged | 1049 | 0.05 | 2.20 (2.10 to 2.29) |
| Female | UK born | No racism | Most disadvantaged | 589 | 0.05 | 2.18 (2.07 to 2.29) |
| Female | Born abroad | No racism | Most disadvantaged | 110 | 0.08 | 2.14 (1.99 to 2.29) |
| Female | UK born | No racism | Middle | 712 | 0.05 | 2.13 (2.03 to 2.23) |
| Female | Born abroad | No racism | Middle | 176 | 0.07 | 2.01 (1.87 to 2.15) |
| Female | Born abroad | No racism | Least disadvantaged | 452 | 0.06 | 1.97 (1.85 to 2.08) |

*Note. Values are model-based marginal predicted means with 95% confidence intervals. SE, standard error; n, number of observations in the stratum. Numeric values are rounded to two decimal places.*

**Supplementary Table 6. Marginal predicted means for hyperactivity/inattention symptoms**

| **Sex** | **Birth status** | **Experience of racism** | **Disadvantage tertile** | **n** | **SE** | **Predicted mean (95% CI)** |
| --- | --- | --- | --- | --- | --- | --- |
| Female | UK born | Racism | Middle | 299 | 0.07 | 4.36 (4.21 to 4.50) |
| Female | UK born | Racism | Most disadvantaged | 211 | 0.07 | 4.29 (4.15 to 4.43) |
| Male | UK born | Racism | Most disadvantaged | 283 | 0.07 | 4.19 (4.05 to 4.33) |
| Female | UK born | Racism | Least disadvantaged | 378 | 0.07 | 4.18 (4.04 to 4.31) |
| Male | UK born | Racism | Middle | 326 | 0.07 | 4.13 (3.99 to 4.26) |
| Male | UK born | Racism | Least disadvantaged | 435 | 0.07 | 3.99 (3.86 to 4.13) |
| Female | UK born | No racism | Most disadvantaged | 589 | 0.06 | 3.84 (3.72 to 3.96) |
| Male | UK born | No racism | Most disadvantaged | 778 | 0.06 | 3.76 (3.64 to 3.87) |
| Male | Born abroad | Racism | Middle | 176 | 0.08 | 3.75 (3.59 to 3.91) |
| Female | Born abroad | Racism | Middle | 98 | 0.08 | 3.74 (3.59 to 3.90) |
| Female | UK born | No racism | Middle | 712 | 0.06 | 3.74 (3.62 to 3.85) |
| Female | UK born | No racism | Least disadvantaged | 1049 | 0.05 | 3.71 (3.61 to 3.82) |
| Female | Born abroad | Racism | Most disadvantaged | 38 | 0.08 | 3.70 (3.54 to 3.87) |
| Male | Born abroad | Racism | Most disadvantaged | 88 | 0.08 | 3.68 (3.52 to 3.84) |
| Male | UK born | No racism | Middle | 818 | 0.06 | 3.66 (3.54 to 3.77) |
| Male | UK born | No racism | Least disadvantaged | 1010 | 0.05 | 3.61 (3.50 to 3.72) |
| Female | Born abroad | Racism | Least disadvantaged | 218 | 0.08 | 3.58 (3.43 to 3.73) |
| Male | Born abroad | Racism | Least disadvantaged | 291 | 0.07 | 3.57 (3.43 to 3.72) |
| Male | Born abroad | No racism | Most disadvantaged | 181 | 0.09 | 3.27 (3.09 to 3.44) |
| Female | Born abroad | No racism | Middle | 176 | 0.07 | 3.26 (3.12 to 3.40) |
| Female | Born abroad | No racism | Most disadvantaged | 110 | 0.08 | 3.23 (3.07 to 3.38) |
| Male | Born abroad | No racism | Middle | 308 | 0.07 | 3.23 (3.09 to 3.36) |
| Female | Born abroad | No racism | Least disadvantaged | 452 | 0.06 | 3.12 (2.99 to 3.24) |
| Male | Born abroad | No racism | Least disadvantaged | 524 | 0.06 | 3.11 (2.98 to 3.23) |

*Note. Values are model-based marginal predicted means with 95% confidence intervals. SE, standard error; n, number of observations in the stratum. Numeric values are rounded to two decimal places.*

**Supplementary Table 7. Marginal predicted means for peer relationship problems**

| **Sex** | **Birth status** | **Experience of racism** | **Disadvantage tertile** | **n** | **SE** | **Predicted mean (95% CI)** |
| --- | --- | --- | --- | --- | --- | --- |
| Male | Born abroad | Racism | Least disadvantaged | 291 | 0.06 | 2.46 (2.35 to 2.58) |
| Female | Born abroad | Racism | Least disadvantaged | 218 | 0.06 | 2.40 (2.28 to 2.52) |
| Male | Born abroad | Racism | Most disadvantaged | 88 | 0.07 | 2.36 (2.22 to 2.51) |
| Female | Born abroad | Racism | Most disadvantaged | 38 | 0.09 | 2.33 (2.14 to 2.51) |
| Male | Born abroad | Racism | Middle | 176 | 0.06 | 2.23 (2.10 to 2.35) |
| Male | UK born | Racism | Least disadvantaged | 435 | 0.06 | 2.11 (2.01 to 2.22) |
| Female | Born abroad | Racism | Middle | 98 | 0.07 | 2.11 (1.98 to 2.24) |
| Male | Born abroad | No racism | Least disadvantaged | 524 | 0.05 | 2.07 (1.97 to 2.17) |
| Female | UK born | Racism | Least disadvantaged | 378 | 0.05 | 1.99 (1.89 to 2.10) |
| Male | UK born | Racism | Most disadvantaged | 283 | 0.06 | 1.99 (1.87 to 2.11) |
| Male | Born abroad | No racism | Most disadvantaged | 181 | 0.07 | 1.97 (1.83 to 2.11) |
| Female | Born abroad | No racism | Least disadvantaged | 452 | 0.05 | 1.96 (1.85 to 2.06) |
| Female | Born abroad | No racism | Most disadvantaged | 110 | 0.07 | 1.86 (1.73 to 2.00) |
| Male | Born abroad | No racism | Middle | 308 | 0.06 | 1.86 (1.75 to 1.97) |
| Male | UK born | Racism | Middle | 326 | 0.06 | 1.85 (1.72 to 1.97) |
| Female | UK born | Racism | Most disadvantaged | 211 | 0.06 | 1.78 (1.67 to 1.90) |
| Female | UK born | Racism | Middle | 299 | 0.06 | 1.77 (1.66 to 1.88) |
| Male | UK born | No racism | Least disadvantaged | 1010 | 0.04 | 1.76 (1.68 to 1.85) |
| Female | Born abroad | No racism | Middle | 176 | 0.06 | 1.70 (1.58 to 1.82) |
| Female | UK born | No racism | Least disadvantaged | 1049 | 0.04 | 1.59 (1.51 to 1.66) |
| Male | UK born | No racism | Most disadvantaged | 778 | 0.04 | 1.56 (1.48 to 1.65) |
| Male | UK born | No racism | Middle | 818 | 0.04 | 1.51 (1.42 to 1.59) |
| Female | UK born | No racism | Most disadvantaged | 589 | 0.05 | 1.38 (1.28 to 1.47) |
| Female | UK born | No racism | Middle | 712 | 0.04 | 1.37 (1.28 to 1.46) |

*Note. Values are model-based marginal predicted means with 95% confidence intervals. SE, standard error; n, number of observations in the stratum. Numeric values are rounded to two decimal places.*

**Supplementary Table 8. Marginal predicted means for emotional symptoms**

| **Sex** | **Birth status** | **Experience of racism** | **Disadvantage tertile** | **n** | **SE** | **Predicted mean (95% CI)** |
| --- | --- | --- | --- | --- | --- | --- |
| Female | Born abroad | Racism | Least disadvantaged | 218 | 0.03 | 4.02 (3.95 to 4.08) |
| Female | UK born | Racism | Least disadvantaged | 378 | 0.03 | 3.90 (3.84 to 3.96) |
| Female | Born abroad | Racism | Most disadvantaged | 38 | 0.04 | 3.90 (3.82 to 3.97) |
| Female | Born abroad | Racism | Middle | 98 | 0.03 | 3.78 (3.71 to 3.84) |
| Female | UK born | Racism | Most disadvantaged | 211 | 0.03 | 3.76 (3.69 to 3.82) |
| Female | UK born | Racism | Middle | 299 | 0.03 | 3.70 (3.64 to 3.75) |
| Female | Born abroad | No racism | Least disadvantaged | 452 | 0.03 | 3.33 (3.27 to 3.38) |
| Female | UK born | No racism | Least disadvantaged | 1049 | 0.03 | 3.23 (3.18 to 3.28) |
| Female | Born abroad | No racism | Most disadvantaged | 110 | 0.03 | 3.14 (3.07 to 3.21) |
| Female | UK born | No racism | Most disadvantaged | 589 | 0.03 | 3.11 (3.06 to 3.17) |
| Female | Born abroad | No racism | Middle | 176 | 0.03 | 3.09 (3.03 to 3.14) |
| Female | UK born | No racism | Middle | 712 | 0.03 | 3.00 (2.95 to 3.05) |
| Male | Born abroad | Racism | Least disadvantaged | 291 | 0.03 | 2.84 (2.78 to 2.91) |
| Male | UK born | Racism | Least disadvantaged | 435 | 0.03 | 2.74 (2.67 to 2.80) |
| Male | Born abroad | Racism | Most disadvantaged | 88 | 0.04 | 2.70 (2.62 to 2.78) |
| Male | Born abroad | Racism | Middle | 176 | 0.03 | 2.60 (2.53 to 2.67) |
| Male | UK born | Racism | Most disadvantaged | 283 | 0.03 | 2.56 (2.50 to 2.62) |
| Male | UK born | Racism | Middle | 326 | 0.03 | 2.51 (2.45 to 2.57) |
| Male | Born abroad | No racism | Least disadvantaged | 524 | 0.03 | 2.13 (2.08 to 2.19) |
| Male | UK born | No racism | Least disadvantaged | 1010 | 0.02 | 2.04 (1.99 to 2.09) |
| Male | Born abroad | No racism | Most disadvantaged | 181 | 0.04 | 1.97 (1.90 to 2.04) |
| Male | UK born | No racism | Most disadvantaged | 778 | 0.03 | 1.90 (1.85 to 1.95) |
| Male | Born abroad | No racism | Middle | 308 | 0.03 | 1.89 (1.82 to 1.95) |
| Male | UK born | No racism | Middle | 818 | 0.03 | 1.82 (1.77 to 1.87) |

*Note. Values are model-based marginal predicted means with 95% confidence intervals. SE, standard error; n, number of observations in the stratum. Numeric values are rounded to two decimal places.*
